# Mediation of the association between multi-morbidity and sleep problems by pain and depressive symptoms among older adults: Evidence from the Longitudinal Aging Study in India, wave- 1

**DOI:** 10.1371/journal.pone.0281500

**Published:** 2023-02-09

**Authors:** T. Muhammad, Trupti Meher, Laeek Ahemad Siddiqui

**Affiliations:** 1 Department of Family & Generations, International Institute for Population Sciences, Mumbai, Maharashtra, India; 2 Department of Bio-Statistics and Epidemiology, International Institute for Population Sciences, Mumbai, Maharashtra, India; Fred Hutchinson Cancer Research Center, UNITED STATES

## Abstract

**Background:**

There could be several possible mechanisms on how chronic conditions relate to sleep problems in older persons; for instance, pain and sleep have a strong link and depressive symptoms are similarly associated with sleep problems. The present study explored whether pain and depressive symptoms are mediators in the relationship between multi-morbidity and sleep problems among older adults.

**Methods:**

Study utilized data from the Longitudinal Aging Study in India (LASI) with a sample of 31,464 older adults age 60 years and above. Multivariable logistic regression along with mediation analysis using Karlson–Holm–Breen (KHB) method was conducted.

**Results:**

A proportion of 14.8% of the participants suffered from sleep problems, whereas, 22.5% and 8.7% of older adults had multi-morbidity and had depressive symptoms, respectively. Also, around 10.3% of older adults reported pain and received no medication for the relief of pain, whereas 29.3% of older adults reported pain and received some type of medication for the relief of pain. Older adults with multi-morbidity had higher odds of suffering from sleep problems [adjusted odds ratio (aOR):1.26, confidence interval (CI):1.10–1.45] than those who had no multi-morbidity. Older adults who reported pain but received no medication for the relief of pain [aOR: 1.90, CI: 1.64–2.22] or reported pain and received medication for the relief of pain [aOR: 1.82, CI:1.62–2.04] and those who had depressive symptoms [aOR: 2.21, CI:1.89–2.57%] had higher odds of suffering from sleep problems compared to those who did not report pain and had no depressive symptoms, respectively. Around 11.2% of the association of multi-morbidity with sleep problems was mediated by pain and 4.3% of such association was mediated by depressive symptoms.

**Conclusion:**

Pain and depressive symptoms were found to mediate the association between multi-morbidity and sleep problems; therefore, reducing pain and depressive symptoms may be considered to improve sleep in older multi-morbid patients.

## Background

The growth of aged population is posing a concern for many countries worldwide, including India. Global estimates predict that by 2040, the share of older population will double compared to their share in 2008 [1]. Similarly, in 2011, India had 103.8 million older adults aged 60 years and above, that is 8.6% of its total population [2] and is expected to have 319 million older adults by 2050, which is 19.5% of its total population [3] As life expectancy rises with each decade, an increasing number of people are suffering with several chronic illnesses [4, 5]. And one of the most common and least-addressed issues associated with ageing is sleep problems [6, 7].

Sleep disturbance is a serious health issue among older adults because poor sleep has been linked to overall health degradation, reduced quality of life, mental health issues, and pre-mature mortality [8–11]. Further, sleep problems affect large number of people around the world [12]. It has been claimed that 30% of adults have some sleep problems, and 10% have chronic sleep problems [13]. Moreover, several studies have shown that older adults have a significant prevalence of sleep problems [14–16]. It is reported that roughly half of older adults have sleeping issue, both initiating and maintaining sleep [17–19]. Furthermore, studies indicate that older adults in both high-income countries (HICs) and low- and middle-income countries (LMICs) suffer from sleep problems to a similar extent [20].

Multiple health-related elements have important role in the development of sleep problems [21]. For instance, hypertension, diabetes and other cardiovascular diseases are strongly associated with sleep problems among older adults [22, 23]. Besides, a study indicated that persons with a heart attack had difficulty in falling asleep; and diabetic patients were observed to be waking up several times per night [24]. Also, it is now commonly acknowledged that multi-morbidity has a strong link to sleep problems [21, 22]. Furthermore, as Tang and colleagues pointed out, pain and health-related anxiety were the strong predictors of sleep problem severity [21, 25]. Moreover, multiple studies found a strong link between depressive symptoms and sleep problems [26, 27]. However, much of the study on the co-occurrence of sleep problems and depression has taken place in HICs and studies on the same in LMICs are scarce due to lack of data [9]. Nevertheless, two recent community-based studies in multiple LMICs indicated a robust link between depression and sleep problems among older adults aged 50 and above [28, 29]. Moreover, multiple studies in India have shown that number of chronic conditions is significantly associated with depressive symptoms in later life [30, 31]. Similarly, a couple of studies suggested a link between chronic illnesses and experiencing pain and the co-occurrence of medical conditions and chronic pain among older adults [32, 33].

**Fig 1:**
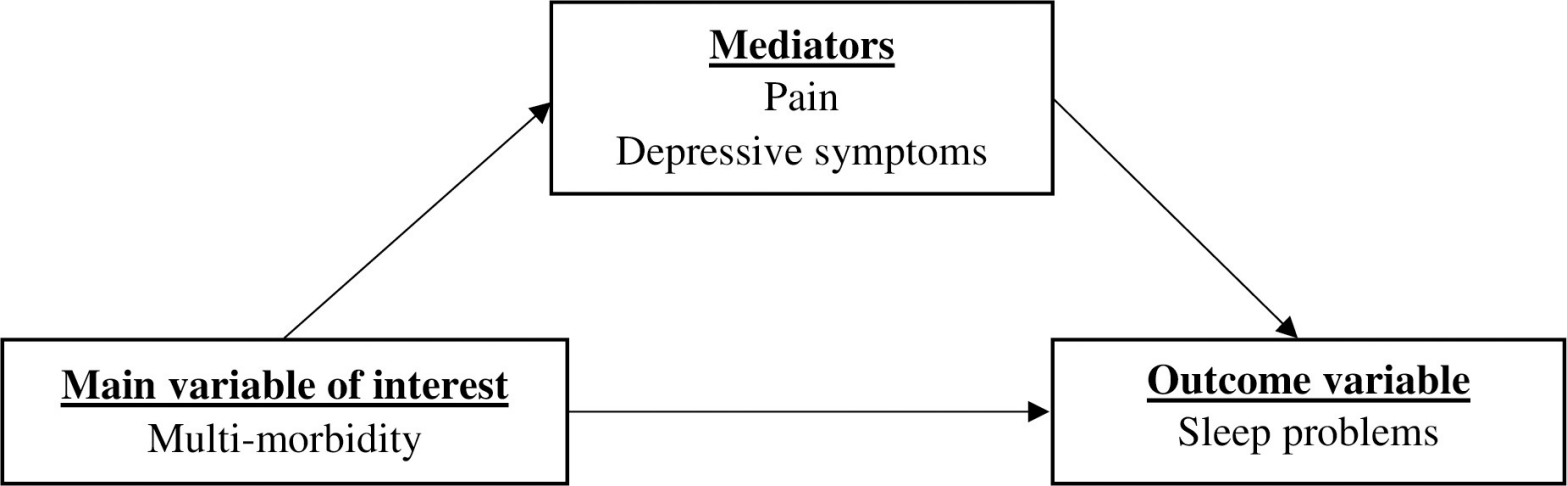
Conceptual model of the study.

Thus, there could be several possible mechanisms on how chronic conditions relate to sleep problems in older persons; for instance, pain and sleep have a strong link [34], and depressive symptoms are similarly associated with sleep problems [27, 35, 36]. Thus, it becomes critical to investigate the role of possible mediating elements of the association between multi-morbidity conditions and sleep problems. The present study aimed to explore whether pain and depressive symptoms are mediators in the relationship between multi-morbidity conditions and sleep problems among older adults. Since appropriate data sources are lacking on India’s growing older population, the current analysis will help fill the gap in the literature among those from low- and middle-income countries on complex health and wellbeing associations, especially in terms of the underlying mechanisms between multi-morbidity and associated sleep problems. This study will also add to the current evidence on the associations of morbidity, pain, depressive symptoms, and sleep among community-dwelling older adults and their care practice in the resource-limited setting of India. Based on the abovementioned literature, a conceptual framework has been developed and summarized in Fig 1.

## Methods

### Study participants

The present study utilizes the individual-level data from the first wave of the Longitudinal Aging Study in India (LASI) conducted during 2017–18 [37]. The LASI is a country-representative longitudinal survey of 72,250 adults aged 45 years and over across all states and union territories of the country that provides vital information on the social, physical, psychological, and cognitive health of the Indian aging population. The LASI survey was conducted through a partnership of the International Institute for Population Sciences (IIPS), Harvard T. H. Chan School of Public Health (HSPH), and the University of Southern California (USC). In the LASI wave 1, the sample selection is based on a multistage stratified cluster sample design, including a three-stage sampling design in rural areas and a four-stage sampling design in urban areas. Individual survey schedule was administered to each consenting respondent in the sampled households. The details of sampling design, survey instruments, and data collection procedures are provided elsewhere [37, 38]. The present study is conducted on the eligible respondents aged 60 years and above. Thus, the total sample size for the present study was 31,464 (15,098 males and 16,366 females) older adults aged 60 years and above.

### Ethics statement

The Indian Council of Medical Research (ICMR) extended the necessary guidance, guidelines and ethical approval for conducting the LASI survey. And all methods were carried out in accordance with those relevant guidelines and regulations. The survey agencies that conducted the field survey for the data collection have collected prior consent (signed and oral) for both the interviews and biomarker tests from the eligible respondents in accordance with Human Subjects Protection.

### Measures

#### Outcome variable

The variable ‘sleep problems’ was assessed with four questions in the LASI survey, adapted from the Jenkins Sleep Scale (JSS-4) [39]. The questions address the respondents’ sleep during the past one month. The items include: “How often do you have trouble falling asleep?” 2) “How often do you have trouble with waking up during the night?” 3) “How often do you have trouble with waking up too early and not being able to fall asleep again?” 4) “How often did you feel unrested during the day regardless of the number of hours of sleep you had?” Response options (referring to the past one month) were “never”, “rarely” (1–2 nights per week), “occasionally” (3–4 nights per week), and “frequently” (5 or more nights per week) [40, 41]. ‘Sleep problems’ was categorized as one if the response was ‘frequently’ for any of the four symptoms and otherwise zero representing no sleep problems [42]. The JSS-4 scale of assessing sleep problems has proved excellent reliability and demonstrated good construct validity [43].” The internal consistency (Chronbach’s alpha value) of JSS-4 scale was 0.87 in this study.

#### Main exposure variables

The variable multi-morbidity was created using the chronic diseases which include hypertension, chronic heart diseases, stroke, any chronic lung disease, diabetes, cancer or malignant tumor, any bone/joint disease. The diseases were self-reported as was assessed through the question “Has any health professional ever diagnosed you with the following chronic conditions or diseases?” And such patient-reported health outcomes are often considered as of high quality than clinical outcome measures [44]. The responses were yes and no, coded as one and zero. Multi-morbidity in this study refers to the presence of two or more of those available chronic diseases.

Secondly, pain was self-reported by older individuals. It was assessed if respondents reported that they were troubled by pain and they required some form of medication or treatment for the relief of pain. The question used to assess the prevalence of pain was “Are you often troubled with pain?” Further, “Do you take any medication or therapy to get relief from the pain?”, the responses were “Yes, analgesics (Oral/ Injectable)”, b. “Yes, therapy(ies)”, c. “Local/external application (Ointment, cream, gel, balm, spray, oil, etc.)” and d. None. Pain was categorized as “no” if the respondent was not troubled with pain, “yes but no medication” if the respondent was troubled with pain and received none of the medications above mentioned, and “yes with medication” if the respondent was troubled with pain and received any type of medications.

Thirdly, the variable ‘depressive symptoms’ was assessed using the CIDI-SF (Short Form Composite International Diagnostic Interview) scale with a cut-off score of three or more on a scale of 0 to 10 [30, 45]. It has three screening questions (based on the presence of dysphoria and/or anhedonia for ≥ 2 weeks during the past 12 months) and seven symptom-based questions. The seven symptoms include loss of interest, feeling tired, loss of appetite, trouble concentrating, feeling of worthlessness, thinking about death and trouble falling asleep [46]. A positive answer to three or more of those lead to the attribution of the label “diagnosed with depressive symptoms”. This scale estimates a probable psychiatric diagnosis of major depression and has been validated in field settings [46]. The scale was validated with well-established cross-cultural applicability especially by non-clinicians in general population surveys and widely used in population-based health surveys [46–48]. Cronbach’s alpha indicated that CIDI-SF has an acceptable reliability (α = 0.68).

#### Other covariates

Self-rated health was assessed by the question, “Overall, how is your health in general? Would you say it is very good, good, fair, poor, or very poor?” It was categorized as good which includes very good, good and fair whereas, poor includes poor and very poor. The activities of daily living (ADL) is a term used to refer to normal daily self-care activities (such as dressing, walking across a room, eating, getting in and out of bed, washing, and using the toilet (Cronbach’s alpha  =  0.87)) The ability or inability to perform ADLs is used to measure a person’s functional status, especially in the case of people with disabilities and the older adults [49, 50]. Instrumental ADLs (IADL) are not necessarily related to the fundamental functioning of a person, but they let an individual live independently in a community. The IADLs include preparing a hot meal, shopping for groceries, making a telephone call, taking medications, doing work around the house or garden, managing money (such as paying bills and keeping track of expenses), and getting around or finding an address in unfamiliar places (Cronbach’s alpha  =  0.88). In this study, older individuals who struggled with any of the six and seven activities in ADL and IADL, respectively for more than three months were labeled as facing difficulty in ADL and IADL and was categorized as no and yes.

Much of the epidemiological research on sleep health has also revealed that socioeconomic, demographic, cultural, and regional differences may alter the quality and amount of sleep among older adults [51, 52]. Thus, the following factors were controlled in the analysis. Socio-demographic variables included age (categorized as 60–69, 70–79 and 80+), sex (male and female), education (categorized as no education, primary education and secondary/higher education), marital status (categorized as married, widowed and others which included separated, divorced and never married), living arrangements (categorized as living alone, with spouse, with spouse and children and others) and work status (categorized as never worked, currently working, not working and retired). The monthly per capita consumption expenditure (MPCE) quintile was assessed using household consumption data, with five quintiles i.e., from poorest to richest. Religion was categorized as Hindu, Muslim, Christian, and Others. Caste was categorized as Scheduled Caste/Scheduled Tribe (SC/ST), Other Backward Classes (OBC), and others. The ‘others’ caste category is identified as having higher social status, mostly belong to upper caste categories [53]. Place of residence was categorized as urban and rural. The regions of the country were categorized as North, Central, East, Northeast, West, and South.

#### Statistical analysis

Descriptive statistics along with results of cross-tabulations are presented in the study. Chi square test was used to determine the significance of the associations between predictor variables and the dependent variable. Additionally, multivariable logistic regression analysis was conducted to find out the association between multi-morbidity and the outcome variable (sleep problems) after adjusting for the selected possible confounders. The association of the two mediators with the outcome was also assessed. The estimates were presented in the form of unadjusted (uOR) and adjusted odds ratio (aOR) along with 95% confidence interval (CI). Model 1 in the logistic regression results provides the unadjusted estimates of sleep problems whereas, Model 2 provides the estimates adjusted for all the selected covariates in Table 1. Individual weights were used to make the estimates nationally representative.

**Table 1:** Sample distribution and rates of sleep problems by background characteristics among older adults (n = 31,464).

Variables	Sample distribution	No sleep problems	Sleep problems	p-value
	Frequency (c w%)	r w%	r w%	
**Sleep problems**				
No	27004 (85.23)			
Yes	4357 (14.77)			
**Multi-morbidity**				<0.001
No	24214 (77.53)	86.69	13.31	
Yes	7250 (22.47)	80.64	19.36	
**Pain**				<0.001
No	18669 (60.41)	89.55	10.45	
Yes but no medication	3694 (10.27)	78.84	21.16	
Yes with medication	8991 (29.32)	78.58	21.42	
**Depressive symptoms**				<0.001
No	28482 (71.33)	87.06	12.94	
Yes	2170 (8.67)	67.62	32.38	
**Age (in years)**				<0.001
60–69	18974 (58.51)	86.38	13.62	
70–79	9101 (30.2)	84.18	15.82	
80+	3389 (11.29)	82.06	17.94	
**Sex**				<0.001
Male	15098 (47.45)	86.12	13.88	
Female	16366 (52.55)	84.44	15.56	
**Marital status**				<0.001
Married	19920 (61.63)	86.17	13.83	
Widowed	10719 (36.2)	83.49	16.51	
Others	825 (2.17)	87.81	12.19	
**Living arrangement**				<0.001
Alone	1622 (5.68)	81.38	18.62	
With spouse	6215 (20.33)	85.97	14.03	
With spouse and children	13465 (40.62)	86.39	13.61	
Others	10162 (33.37)	84.04	15.96	
**Educational status**				<0.001
None	16889 (56.52)	84.51	15.54	
Primary	5840 (17.5)	87.6	15.34	
Secondary/higher	8735 (25.98)	86.73	12.67	
**Work status**				<0.001
Never worked	8784 (26.43)	85.76	14.24	
Not working	10990 (36.45)	82.42	17.58	
Working	8997 (29.87)	88.25	11.75	
Retired	2693 (7.25)	84.92	15.08	
**Self-rated health**				<0.001
Good	23685 (75.79)	88.31	11.69	
Poor	7113 (24.21)	76.1	23.9	
**ADL difficulty**				<0.001
No	24642 (76.23)	87.75	12.25	
Yes	6694 (23.77)	77.2	22.8	
**IADL difficulty**				<0.001
No	17449 (51.64)	89.11	10.89	
Yes	13846 (48.36)	81.1	18.9	
**MPCE quintile**				0.113
Poorest	6484 (21.7)	85.44	14.56	
Poorer	6477 (21.71)	84.91	15.09	
Middle	6416 (20.95)	85.49	14.51	
Richer	6170 (19.19)	86.08	13.92	
Richest	5917 (16.45)	84.07	15.93	
**Caste**				<0.001
SC/ST	10313 (27.03)	83.77	16.23	
OBC	11886 (45.23)	85.76	14.24	
Others	9265 (27.74)	85.81	14.19	
**Religion**				<0.001
Hindu	23037 (82.22)	85.4	14.6	
Muslim	3731 (11.28)	84.36	15.64	
Others	4696 (6.5)	84.65	15.35	
**Place of residence**				<0.001
Urban	10739 (29.45)	87.23	12.77	
Rural	20725 (70.55)	84.42	15.58	
**Region**				<0.001
North	5812 (12.59)	85.52	14.48	
Central	4262 (20.95)	82.02	17.98	
East	5757 (23.64)	85.89	14.11	
Northeast	3752 (2.97)	92.12	7.88	
South	7578 (22.68)	86.64	13.36	
West	4303 (17.17)	85.03	14.97	
**Total**	31,464	85.23	14.77	

Notes: Frequencies are un-weighted; c w%: weighted column percentages to account for survey design and provide national population estimates; r w%: weighted prevalence estimates (row percentages); p-values are based on Chi-square test; ADL: Activities of daily living; IADL: Instrumental activities of daily living; MPCE: Monthly per capita consumption expenditure

Additionally, the total effect in the observed association was divided into direct effects (the association of multi-morbidity with sleep problems controlling for all the covariates) and indirect or mediating effects (the association of multi-morbidity with sleep problems through pain and depressive symptoms) using Karlson–Holm–Breen (KHB) method [54, 55]. The KHB method is a recently developed method for assessing mediating effects that allow total effects to be divided into direct and indirect (i.e., mediational) effects for both discrete and continuous variables. Contrary to other decomposition methods, the KHB-method provides unbiased decomposition results [56]. The mediation percentage (the indirect effect divided by the total effect) is interpreted as the percentage of the association explained by the mediator variable. All statistical models were adjusted for various predictors, including age, sex, education, marital status, living arrangements, work status, SRH, ADL/IADL difficulty, MPCE quintiles, religion, caste, place of residence and regions. Variance inflation factor (VIF) was generated in STATA 15 [57] to check the multicollinearity in the variables used [58, 59]. The statistical analysis was performed using Stata 15.1 [57].

## Results

Table 1 presents the sample characteristics and prevalence estimates of sleep problems among older adults in the study. 14.8% of the participants reported sleep problems, whereas, 22.5%, and 8.7% of older adults had multi-morbidity and depressive symptoms, respectively. Also, around 10.3% of older adults reported pain and received no medication for the relief of pain, whereas 29.3% of older adults reported pain and received some type of medication for the relief of pain. Further, a proportion of 11.3% of older adults were aged 80+ years in the study. Out of total sample, 52.6% were women, 36.2% were widowed and 5.7% were living alone. More than half of the study population (56.5%) had no formal education, whereas, 29.9% of older adults were currently working. Besides, 24.2% of older adults had a poor SRH and a proportion of 23.8% and 48.4% of older adults had at least one difficulty in ADL and IADL, respectively.

Further, 19.4% of older adults who had multi-morbidity suffered from sleep problems compared to 13.4% of those who had no multi-morbidity. Similarly, 21.4% of those who reported pain and received some type of medication for the relief of pain suffered from sleep problems against only 10.5% of those who did not report pain. On the other hand, 21.2% of those who reported pain and received medication suffered from sleep problems. Also, a higher proportion of those who had depressive symptoms (32.4%) suffered from sleep problems than those who had no depressive symptoms (12.9%).

Table 2 presents the results from the multivariable regression analysis of sleep problems for different explanatory variables. Adjusted estimates show that older adults with multi-morbidity had higher odds of suffering from sleep problems [aOR: 1.26, CI: 1.10–1.45] than those who had no multi-morbidity. Similarly, older adults who reported pain but received no medication for the relief of pain [aOR: 1.90, CI: 1.64–2.22] or reported pain and received medication for the relief of pain [aOR: 1.82, CI: 1.62–2.04] and those who had depressive symptoms [aOR: 2.21, CI: 1.89–2.57%] had significantly higher odds of suffering from sleep problems compared to those who did not report pain and had no depressive symptoms, respectively.

**Table 2:** Logistic regression estimates of sleep problems by socioeconomic and health characteristics among older adults (n = 30,578).

Variables		Model 1	Model 2
		uOR (95% CI)	aOR (95% CI)
**Multi-morbidity**	No	Ref.	Ref.
	Yes	1.40 (1.23–1.58)	1.26 (1.10–1.45)
**Pain**	No	Ref.	Ref.
	Yes but no medication	2.30 (1.99–2.65)	1.90 (1.64–2.22)
	Yes with medication	2.34 (2.09–2.61)	1.82 (1.62–2.04)
**Depressive symptoms**	No	Ref.	Ref.
	Yes	2.84 (2.45–3.29)	2.21 (1.89–2.57)
**Age (in years)**			1.00 (0.99–1.01)
**Sex**	Male		Ref.
	Female		0.96 (0.83–1.11)
**Marital status**	Married		Ref.
	Widowed		0.58 (0.35–0.96)
	Others		0.48 (0.27–0.85)
**Living arrangement**	Alone		Ref.
	With spouse		0.47 (0.27–0.80)
	With spouse and children		0.47 (0.28–0.81)
	Others		0.88 (0.68–1.14)
**Education (in years)**			1.01 (0.99–1.02)
**Work status**	Never worked		Ref.
	Not working		1.18 (1.02–1.37)
	Working		0.96 (0.81–1.14)
	Retired		1.20 (0.94–1.53)
**Self-rated health**	Good		Ref.
	Poor		1.62 (1.44–1.82)
**ADL difficulty**	No		Ref.
	Yes		1.31 (1.14–1.50)
**IADL difficulty**	No		Ref.
	Yes		1.34 (1.18–1.52)
**MPCE quintile**	Poorest		Ref.
	Poorer		1.08 (0.93–1.27)
	Middle		1.02 (0.87–1.20)
	Richer		0.97 (0.83–1.14)
	Richest		1.09 (0.92–1.29)
**Religion**	Hindu		Ref.
	Muslim		1.11 (0.94–1.30)
	Others		0.99 (0.82–1.18)
**Caste**	SC/ST		Ref.
	OBC		0.84 (0.74–0.97)
	Others		0.85 (0.73–0.99)
**Place of residence**	Urban		Ref.
	Rural		1.17 (1.03–1.33)
**Region**	North		Ref.
	Central		1.16 (0.99–1.37)
	East		0.78 (0.67–0.90)
	Northeast		0.45 (0.36–0.58)
	West		0.87 (0.74–1.02)
	South		0.96 (0.81–1.14)

Notes: uOR: unadjusted odds ratio; aOR: OR adjusted for age, sex, marital status, living arrangements, education, work status, self-rated health, ADL/IADL difficulty, MPCE quintile, religion, caste, place of residence and regions; ADL: Activities of daily living; IADL: Instrumental activities of daily living; MPCE: Monthly per capita consumption expenditure

Table 3 provides the mediation effects of pain and depressive symptoms in the association of multi-morbidity and sleep problems among older adults. Direct effect estimates showed that older adults who were multi-morbid had higher odds of sleep problems despite the mediation effects of pain [aOR: 1.39, CI: 1.28–1.50] and depressive symptoms [aOR: 1.46, CI: 1.35–1.58] compared to those who had no multi-morbidity. Around 11.2% of the association between multi-morbidity and sleep problems was mediated by pain and 4.3% was mediated by depressive symptoms.

**Table 3:** Direct and indirect effects of multi-morbidity on sleep problems through pain and depressive symptoms (n = 30,578).

	Total effect	Natural direct effect	Natural indirect effect	Percent effect mediated
Outcome	aOR (95% CI)	aOR (95% CI)	aOR (95% CI)	%
Multi-morbidity on sleep problems				
via pain	1.44 (1.33–1.56)	1.39 (1.28–1.50)	1.04 (1.03–1.05)	11.20
Multi-morbidity on sleep problem				
via depressive symptoms	1.46 (1.35–1.58)	1.44 (1.33–1.55)	1.02 (1.01–1.02)	4.25

Notes: aOR: OR adjusted for age, sex, marital status, living arrangements, education, work status, self-rated health ADL/IADL difficulty, MPCE quintile, religion, caste, place of residence and regions

## Discussion

Chronic illnesses and sleep problems are common among older population [60]. In this study of a sample of community-dwelling older adults aged 60 years and above, around 15 percent of the elderly people in India reported sleep problems, which is consistent with the findings of Koyanagi et al. (2014) [29]. Moreover, the study has found that nearly 22.5 percent of the older adults were suffering from multi-morbidity. On the other hand, older individuals with multi-morbidity have shown higher odds of sleep problems than their counterparts. This finding is in line with other studies that have documented an association between number of chronic diseases and sleep problems among older population [29, 61]. A cross-sectional study of community-dwelling older adults aged 75 and above has found sleep problems among 40 percent of the individuals with multi-morbidity [62]. Another longitudinal study conducted in South Korea has reported that the number of physical disorders at baseline is associated with the incidence and prevalence of sleep problems [63].

The presence of sleep-disordered breathing in illnesses including chronic lung disease, diabetes, and stroke, as well as symptoms of the chronic disorders themselves, can explain this link between multi-morbidity and sleep problems [64, 65]. A potential biological mechanism suggests that some chronic conditions may induce changes in sleep-controlling brain areas and neurotransmitters, and the medicines used to manage chronic conditions have an impact on sleep quality [66]. Psychological distress and anxiety associated with chronic conditions can also contribute to sleep problems [29, 67]. Moreover, chronic illnesses may have an impact on sleep as a result of economic burden. Individuals with multi-morbidity incur significant long-term medical expenses, putting a significant strain on family and personal finances [68, 69]. Furthermore, chronic illnesses have a detrimental impact on family relationships and social interactions, contributing to more negative life events and stressful emotions [70]. Individuals suffering from multi-morbidity experience more negative life events than healthy people. Previous studies have indicated that negative and stressful life events are positively associated with sleep problems [71, 72].

Furthermore, the findings of the present study verified the hypothesis that there was a mediating effect of pain and depressive symptoms in the association between multi-morbidity and sleep problems. In addition, mediation analysis suggested that pain mediated 12.4 percent of the total effect, whereas the effect of multi-morbidity mediated by depressive symptoms on sleep problems was 4.3 percent. This finding indicated that our mediators played a role in explaining the relationship between multi-morbidity and sleep problems among older population. This corroborates with prior research that has shown that pain caused by chronic diseases and psychological disorders like depressive symptoms and anxiety have an impact on sleep [73–75]. However, as documented, the effect of chronic illnesses on mental health may depend on sleep, since restful sleep may allow individuals to cope with illness adaptively [60]. Thus, the current findings shed light on many intriguing relationships between the physical and mental illnesses and their associated symptoms which suggest the need for future research.

The mediating effect of pain on the relationship between multi-morbidity and sleep problems in our study may be because pain is one of the symptoms of chronic illnesses. Chronic conditions like bone/joint related diseases, cancer, etc., are often accompanied with pain sensations [76, 77]. Chronic diseases are long-course diseases; therefore, the discomfort induced by chronic conditions is also long-lasting. Pain caused by physical discomfort reduces individuals’ quality of life and causes unpleasant feelings and emotional disturbances, all of which contribute to sleep problems. Individuals with multi-morbidity are more likely to experience a greater degree of pain [78]. They may experience anxiety, irritability, and other negative emotions as a result of the long-term accumulation of pain, which increases the likelihood of sleep problems significantly. Nonetheless, greater pain severity is associated with a higher degree of sleep problems [79]. Furthermore, a study by Peters et al. (2018) has found sleep disturbances in nearly 60 percent of the depressed older adults [80]. Another longitudinal study in Japan found that around 80 percent of older individuals suffering from depressive symptoms complained of sleep problems, both in terms of quality and quantity [81]. According to Chen et al. [42], sleep time is influenced by numerous neurotransmitter systems, including norepinephrine and 5-hydroxytryptophan, which are also important for mental and emotional functioning [82].

Although it included a large sample which is nationally representative and considered a large number of potentially confounding variables in the analysis, the current study shows some limitations that need to be highlighted in order to make careful interpretation of the findings. Importantly, the cross-sectional design of the study limited any causal inferences from being made on the observed associations. Also, self-report of chronic conditions and pain may bias the current findings due to under or overestimation. As mentioned earlier, past literature has suggested relationships between factors such as chronic conditions, pain, and depressive symptoms and sleep problems to be bi-directional or have reverse-causality. Therefore, a prospective or longitudinal design will be essential to examine the respective associations and to help elucidate the direction of the relationship between sleep and health. Further, stressful and emotional events and medications can have substantial impact on sleep among older adults which could not be evaluated in the current study. Future investigations including these aspects might be useful in exploring the underlying mechanisms between multi-morbidity and sleep problems. Further information regarding the factors associated with sleep such as watching television, using a computer before bedtime as well as duration and frequency of sleep might also be needed to gain further insight into the complex associations among physical, mental and sleep health in older individuals.

Although there was no evidence of multicollinearity in the variables used in the current study, one of the items in the CIDI-SF is "trouble with sleep or difficulty falling asleep", which may conceptually overlap with the outcome of interest, sleep problems. Thus, the observed association between depressive symptoms and the outcome, and depressive symptoms to be a mediator of the association of interest maybe subject to bias. Similarly, the lack of precision with the measurement of pain may be why the mediating effect is so minimal in the current study. The chronicity and severity of pain need to be collected in future rounds of LASI survey which can provide more explanation on this aspect. Furthermore, the study is cross-sectional which makes mediation very challenging to examine. A key assumption of mediation analyses is the ability to determine temporal ordering of variables. In some cross-sectional analyses, ordering can be argued. However, in this study, disentangling these variables is very challenging as sleep problems can cause pain/depressive symptoms. Mediation in this study could cause highly biased results that may not be replicable in prospective studies. Given this, it’s difficult to make clinical suggestions. Further explanations are needed to support the directionality proposed. Final key limitation is the self-reported measure of chronic conditions which is likely to undercount the true prevalence and the use of "ever diagnosed" for chronic conditions which may also include if an older adult had stroke or cancer 5 or 10 years ago and has now fully recovered, with no signs or symptoms. These limitations need to be addressed in future studies.

## Conclusion

In conclusion, the present study indicated that a considerable proportion of older individuals with multi-morbidity have sleep problems. Given the higher prevalence of both sleep problems and multi-morbidity in India, health promotion programmes should be designed to enhance and promote healthy lifestyles in order to reduce multi-morbidity and sleep problems. Furthermore, pain and depressive symptoms were found to play mediating roles in the association between multi-morbidity and sleep problems. Therefore, reducing pain and depressive symptoms might improve sleep quality in older adults with multi-morbid conditions. Moreover, when intervening for older individuals with chronic conditions and sleep problems, pain and depressive symptoms could be considered as the primary intervention targets. Future research is needed to determine the optimal treatment choices for comorbid sleep problems, particularly in resource-constrained settings.
